# Microhardness of bi-antibiotic-eluting bone cement scaffolds

**DOI:** 10.1186/2194-0517-1-3

**Published:** 2012-10-08

**Authors:** Mrinal Musib, Jeremy Jones, Karunesh Chakote, Westley Hayes, Subrata Saha

**Affiliations:** Department of Orthopaedic Surgery and Rehabilitation Medicine, SUNY Downstate Medical Center, Brooklyn, NY 11203 USA

**Keywords:** Hardness, PMMA, Bi-antibiotic-impregnated bone cement, Elution of drugs, Mechanical properties

## Abstract

Bi-antibiotic-impregnated bone cements (BIBCs) are widely used in orthopaedics as a prophylactic agent (depot) to address post-surgical infections. Although hardness is widely considered a viable index to measure the integrity of the cement structure, there are few specific studies involving changes in hardness characteristics of BIBCs post elution of high doses of two widely used antibiotics: tobramycin and gentamicin. Increased doses of antibiotics and increased duration of elution may also decrease the hardness of polymethyl methacrylate (PMMA) bone cement, thus increasing the chances of shattering, scratching, and deformation.

In this project, we have investigated the changes in surface hardness of five different antibiotic-loaded specimens: 0.5 g tobramycin and 0.5 g gentamicin together, 1 g tobramycin, 1 g gentamicin, 5 g tobramycin and 5 g gentamicin together, and 10 g tobramycin (each added to 40 g of PMMA), post elution for various time periods (1, 3, and 21 days). The effect of hydration on the hardness of bone cement was studied to replicate *in vivo* conditions. The micro-indentation tester (Buehler m5103) was utilized to determine if the increased antibiotic loads would compromise the integrity of the bone cement matrix.

The results demonstrated that the amount of drug initially incorporated determined the hardness of the cement post elution. As compared to the control (no antibiotic), specimens containing 1 and 10 g of antibiotic exhibited over 50% and 73% decrease in hardness, respectively. The different treatment durations (post 1 day) as well as the hydration conditions had insignificant effect on the hardness of the cement.

**Electronic supplementary material:**

The online version of this article (doi:10.1186/2194-0517-1-3) contains supplementary material, which is available to authorized users.

## Background

Coming to prevalence around half a century ago, bone cements are widely used in total joint replacements or total joint arthroplasties (TJAs). Antibiotic-impregnated bone cement (AIBC) is widely used to anchor the prosthetic device to the surrounding bone, thus providing mechanical strength to the bone-implant structure (Armstrong et al. [2002]; [Lewis 2009]; [Saha and Pal 1984]; Anagnostakos et al. [2006]; [Buchholz and Engelbrecht 1970]; [Hanssen 2004]; Persson et al. [2006]). Although these procedures are highly successful, there is a possibility of infection which varies from 0.5% to 3% of all surgeries performed (Jiranek et al. [2006]). If the prosthesis or the surrounding area becomes infected, there are usually two options, one being exchange arthroplasty, where the original prosthesis is removed through debridement and replaced. The more prevalent and more successful option, however, is a two-stage revision arthroplasty. In a two-stage revision arthroplasty, the patient undergoes two surgeries: first, an antibiotic-impregnated polymethyl methacrylate (PMMA) spacer is implanted at the site of infection that serves to hold the joint in place, providing a direct and quick therapeutic effect and acting as a temporary prosthesis. In the second, an aseptic prosthesis replaces the spacer. In many cases, the AIBC is used as a means of prophylaxis (Cui et al. [2007]; Masri et al. [1998]). The antibiotic leaches out from the cement matrix and provides protection against pathogens (He et al. [2002]; Hendriks et al. [2004]; Hofmann et al. [2005]; Hsieh et al. [2005]; Joseph et al. [2003]).

Recently, there have been instances of antibiotic-resistant pathogens, and to counter such organisms, clinicians are increasingly using two different broad-spectrum antibiotics (tobramycin and gentamicin) at high doses (Durbhakula et al. [2004]; Greene et al. [1998]; Hsieh et al. [2006]; Klekamp et al. [1999]; Penner et al. [1996]; Lautenschlager et al. [1976]; Dunne et al. [2008]). Unfortunately, at higher doses, antibiotics may deteriorate the mechanical properties of PMMA (Ong et al. [2009]; Baleani et al. [2003]; [Lewis 2008]; Simpson et al. [2005]; Baleani et al. [2008]). Studies indicate that the elution of antibiotics involves a quick burst release from the cement surface that is exposed to blood/body fluids within the first few hours followed by a slow release of antibiotics over several weeks. During longer elution durations, more pores/channels are created within the matrix, thus facilitating further release of antibiotics but nonetheless compromising the cement structure ([Anagnostakos and Kelm 2009]).

Hence, studying and understanding the biomechanical properties as a result of increased antibiotic doses and elution times have become more important and relevant. Although the hardness of bi-antibiotic-impregnated bone cement (BIBC) pre- and post elution is of much significance, there are few specific studies on this important biomechanical characteristic. As more pores are created either through increased elution times or antibiotics, the PMMA cement becomes more susceptible to deformation, shattering, and scratching, destroying the prosthesis and submitting the patient to more surgeries, difficulties, and inconvenience. This study on hardness vs. elution times and increased antibiotic dosages demonstrates the increased susceptibility to these problems.

The purpose of this study was twofold: tests were done to discern any significant change in hardness of Simplex P® bone cement after increased elution times of gentamicin and tobramycin (1, 3, and 21 days) and after increased doses. Previous investigators have extensively studied the elution of antibiotics from bone cement and corresponding changes in biomechanical properties, but hardness tests are not well investigated. Past researchers have primarily tested the effects of vacuum mixing versus hand-mixing (Zivic et al. [2012]). Hand-mixing increases the amount of pores present in PMMA; consequently, deformation/hardness tests provided evidence that hardness increased as the PMMA cement was vacuumed for longer periods of time. To the best of our knowledge, this is the first study involving the investigation of change in hardness of BIBC following elution of varying doses (1 to 10 g) of tobramycin and gentamicin at varying time periods (1 day to 3 weeks).

## Results

Tests were conducted to study changes in hardness of AIBC following increased elution times and antibiotic loads. The hypothesis was that with increased elution times and antibiotic loads, hardness would decrease and show a negative correlation and linear relationship. To simulate actual *in situ* conditions, wet samples were also examined. No significant differences were observed between the hardness values of wet vs. dry samples (*p* < 0.05). The hypothesis was proved correct for increased antibiotic loads. For all elution times, a strong negative relationship was observed, with no Pearson correlation coefficient falling below −0.7 (in absolute value). Also, all *p* values were <0.05, further signifying a significant correlation.

**Figure 1:**
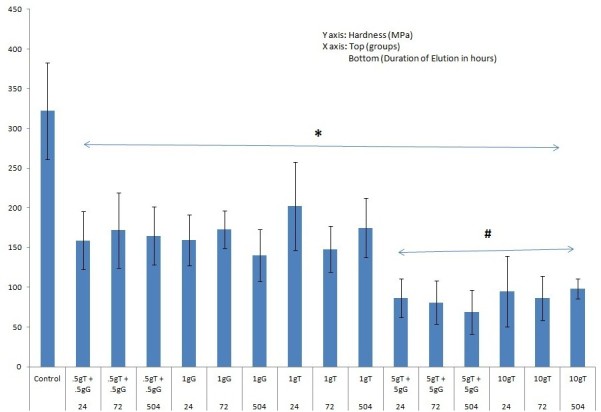
**Graph showing hardness values (** ***Y*** **axis) and the various groups/time periods (** ***X*** **axis).** All data are mean ± SD.

As shown in Figure 1, there was a significant decrease (*p* < 0.05) in hardness between the control and all AIBC specimens at all time points. All data are mean ± standard deviation (SD). The ‘asterisk’ indicates *p* ≤ 0.05 (significant difference between control and individual treatments for all groups), and the ‘number sign’ indicates *p* ≤ 0.05 (significant difference in hardness values was observed between the low dose (1 g) and high dose (10 g) of antibiotics when compared to each other and to the control). Interestingly, there was no time-dependent significant decrease in hardness values beyond 24 h for any group. Also, at any particular time period, the groups exhibited dose-dependent hardness values. For group A, at 1 day, there was a 50% decrease in hardness as compared to the control, which is significantly different from specimens treated for 21 days. Likewise, the other groups exhibited a similar surface hardness profile at all time periods. However, when we consider groups D and E that have a higher antibiotic/PMMA ratio, they exhibited a greater decrease in hardness values. For example, specimens of group D at 1 day exhibited over 73% decrease in hardness as compared to the control, which increased to 78% at 21 days. Group E also exhibited a significant decrease in the hardness value (70%), which does not change significantly with time. A plausible explanation may be that the hardness testing was performed on the surface, and most of the drug present on the surface had already eluted by 24 h. This, however, does not change with increasing elution time. It is only dependent on the initial amount of drug that was incorporated. Few disparities that were observed in this otherwise general trend may be attributed to varying surface properties of the AIBC, even among specimens of the same group.

Table 1 shows the change in hardness values (MPa) at various elution time periods and increasing antibiotic doses. The control had a hardness of 321.9 ± 61.1 MPa, which was significantly greater than the hardness of all specimen groups. Moreover, for the groups that had the same antibiotic/PMMA ratio, we did not observe a significant difference in hardness values for all the time periods. There was a significant decrease in hardness values when the amount of drug was increased from 1 to 10 g for every 40 g of PMMA. Even within the same time periods, the specimen groups containing 1 g of antibiotic exhibited over a 50% decrease in hardness as compared to the control followed by a further 23% decrease when the antibiotic content was increased to 10 g. When two different antibiotics were mixed in place of a single antibiotic (same weight), there was no significant change in hardness values. There was no relationship or correlation between the hardness and increased elution periods post 1 day.

**Table 1:** **Hardness values for various groups and percentage decrease compared to control at various time periods**

Group	24 h (1 day)	72 h (3 days)	504 h (21 days)
	MPa (%)	MPa (%)	MPa (%)
0.5 g G + 0.5 g T	159.0 ± 36.8 (50.6)	171.8 ± 47.5 (46.6)	164.8 ± 36.8 (48.8)
1 g G	159.9 ± 31.9 (50.3)	172.6 ± 23.5 (46.4)	140.2 ± 32.6 (56.4)
1 g T	202.7 ± 55.4 (37.0)	147.8 ± 28.9 (54.1)	174.9 ± 37.5 (45.7)
5 g G + 5 g T	86.7 ± 24.2 (73.1)	81.0 ± 27.4 (74.8)	69.0 ± 27.5 (78.6)
10 g T	95.0 ± 44.3 (70.5)	86.5 ± 27.7 (73.1)	98.3 ± 12.7 (69.5)

G, gentamicin; T, tobramycin.

### Effect of hydration on hardness of PMMA specimen

**Figure 2:**
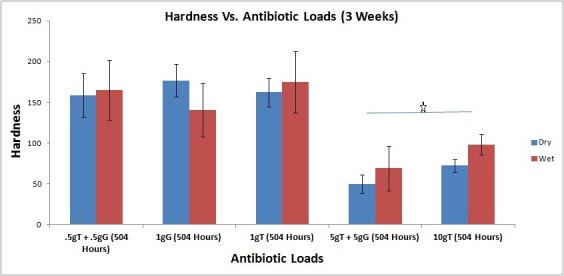
**Hardness values (** ***Y*** **axis) of dry and wet specimens post elution of bi-antibiotics.** All data are mean ± SD. The star indicates *p* ≤ 0.05 (significant difference between low and high antibiotic dose).

There was no significant change in hardness characteristics as a result of hydration at the various time periods of 1, 3, and 21 days post elution. Shown in Figure 2 are the hardness values of the various groups at 3 weeks. Both dry and wet specimens exhibited similar hardness values. This may be attributed to the fact that the curing process is accomplished within the first few hours of the fabrication of the AIBC specimens. Beyond the first few hours, there is very little change in volume or other physicochemical properties of the antibiotic-eluting bone cement (AIBC) specimens.

## Discussion

This study demonstrates that as the amount of antibiotic loaded in the bone cement increases, there is a decrease in its surface hardness post elution of antibiotics. The decrease in hardness may be attributed to the fact that the samples with 10 g of antibiotic content (groups D and E) make up a higher percentage of the bone cement matrix. Leaching of antibiotics creates pores or cavities within the AIBC structure, thus weakening it. Therefore, even though increasing antibiotics might lead to a better therapeutic effect, the structural integrity of the cement may be compromised.

Increased elution time, after 1 day, however, does not seem to have the same effect. Although extending the elution process allows for more antibiotics to elute, via the pores in the matrix and along the surface, it is not enough to demonstrate that the cement is more prone to shattering, scratching, or deformation. However, 3 weeks is the maximum elution period tested in this project, and it does not mean that higher elution times will not decrease hardness, but with regard to the data exhibited in the study, the length of time the load-bearing PMMA bone cement is allowed to stay within the patient before the second surgery of two-stage revision arthroplasty is not detrimental to its structural integrity. This exemplifies an important point. A decrease in hardness, which signifies an increased chance of deformation and shattering of the prosthesis, is caused by increased antibiotic loads. However, increasing elution times, as per these data, does not compromise the structural integrity significantly.

**Figure 3:**
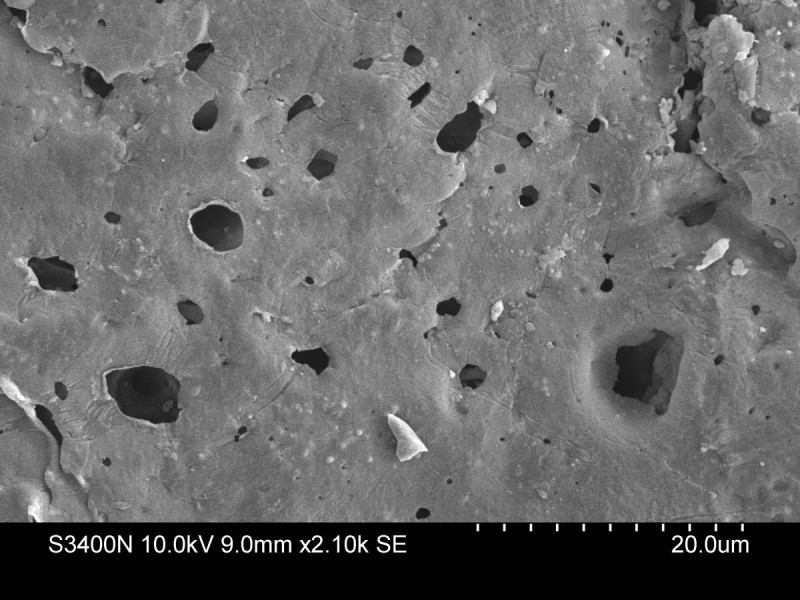
**Creation and distribution of pores/channels created as a result of inhomogeneous distribution of antibiotics.**

Tests were conducted only on the surface of the cement, indicating that during the burst elution phase, the surface hardness of AIBC deteriorates, thus weakening the structural integrity of the cement. Future studies should test for the change in hardness not only along the surface, but also in the interior of the bone cement matrix. This might help to further enforce the theory behind the burst phase phenomenon. Care should be taken in mixing the antibiotics and the PMMA as homogenously as possible, lack of which may result in uneven distribution of the antibiotics on the surface as well as within the core of the matrix. In such cases, the physical properties of the specimen may vary even within the same group. Figure 3 shows the presence of pores/channels on the surface of the scaffold and their distribution.

Future studies will involve smaller increases in the antibiotic loads. Further investigations need to be pursued to understand the change in hardness prior to 24 h. This may be accomplished by having few time points within the 24-h period itself. This will also provide information about the initial burst release phenomenon.

In this experiment, the samples were hydrated to simulate *in situ* conditions; however, there have been other experiments where bone tissue hardness was tested in living humans. A study by Diez-Perez et al. ([2010]) analyzed bone tissue *in vivo*; in addition, their experimentation was to discern if there is a bone hardness difference between osteoporotic patients and patients without this ailment. Similar to our own investigation, they used a micro-indentation machine and used a reference point indentation, and with the use of a local anesthetic, a patient was tested for bone hardness. Unlike the machine used in our experiment, indentations were measured by a specific amount of force applied to the bone and the length of the indentation made was recorded. Although their results indicated that patients with osteoporosis were in fact weaker than patients without the disease, they did not find any correlation between the bone hardness and the age of the patient.

More recently, Zivic et al. ([2012]) studied deformation characteristics of hand-mixed cement and vacuum-mixed cement. Hand-mixing increased the amount of pores, compromising the structural integrity of the bone cement and being more prone to deformation (i.e., decreased hardness). After vacuuming, there was a decrease in the amount of pores; consequently, hardness increased.

A more holistic understanding of PMMA bone cement and its mechanical properties can be found by incorporating not just compression and fatigue studies, but also hardness studies on the same samples. Moreover, comparing these properties against the two types of preparation, vacuum vs. hand-mixing, leads to a comprehensive acumen that will allow for the most optimally fabricated AIBC for patients who undergo TJAs.

Appropriate hardness is one of the important criteria that determine the long-term success of AIBC scaffolds post arthroplasty. The release of antibiotics from such matrices and the corresponding change in biomechanical characteristics (including hardness) are indicators of the longevity of AIBC. The purpose of bone cement in the first place is to anchor the bone as well as to distribute the load and prevent stress shielding. Antibiotics are added as a therapeutic agent to counter potential infections post surgery. The hardness of the bone depends on its location in the body as well as its load-bearing properties, and the biomechanical properties of the bone cement should be similar to those of the adjacent bone. Hengsberger et al. ([2001]) calculated the hardness to be from 0.6 ± 0.11 GPa for compact bone to 1.1 ± 0.17 GPa for trabecular bone. Zysset et al. ([1999]) found it to be between 0.234 GPa for trabecular bone and 0.76 GPa for compact bone. These values are in close agreement to our own results. The control specimen (no antibiotics) had a hardness of 321.9 ± 61.1 MPa, while the other extreme of 10 g of antibiotic had a hardness of 72.2 ± 7.7 MPa.

Most bone cement samples exhibited a burst release during the first 3 h followed by sustained release over the rest of the duration of release. About 4.3% of gentamicin present in the matrix was released from the samples containing gentamicin alone as compared to 18.6% for samples containing tobramycin alone.

Following the burst release of antibiotics during initial few hours of elution, all the antibiotics present at the surface of the scaffold elute, resulting in the creation of pores/channels as revealed by scanning electron microscopy. This results in the decrease of surface hardness. The greater the initial dose and volume fraction of the antibiotics, the greater is the decrease in the hardness. Further investigations need to optimize the antibiotic dose that will not significantly decrease the surface hardness below a clinically acceptable limit.

There are few points to consider when studying biomechanical characterization of AIBCs: (a) homogeneity of distribution of the antibiotics, particularly on the surface; (b) volume ratio of the antibiotics to PMMA; and (c) varying surface porosities of the AIBC. To the knowledge of the authors, there have been no specific studies on the hardness of PMMA bone cement with regard to loaded bi-antibiotics and long elution times. Further studies on hardness should be initiated as they provide another means of testing the strength of the bone.

## Conclusions

Hardness of AIBC is an important biomechanical parameter that helps determine the long-term success of implant devices. As a higher antibiotic dose compromises the hardness as well as other mechanical properties, the dose of such additives should be optimized. The results demonstrate that (a) there was no significant difference in the hardness values between wet and dry specimens and (b) there was minimal correlation between the duration of elution and the hardness of the specimens, although there was a significant decrease in hardness as the antibiotic concentration was increased (*p* ≤ 0.05). Further experimentation would have to be done to investigate the hardness against elution kinetics between hand-mixed specimen and vacuum-mixed specimen. This study has added necessary information illustrating that hardness is compromised as antibiotic concentrations increase; this allows future prosthesis operations to adjust their antibiotic concentrations to attain the best antibiotic therapy without compromising the hardness significantly.

## Methods

All tests were conducted on Simplex P® (Stryker/Howmedica Osteonics, Limerick, Ireland) bone cement with/without the antibiotic(s). The antibiotics, tobramycin and gentamicin (as salts), were acquired from Sigma (cat. #T1783-500MG for tobramycin and cat. #G3632-10G for gentamicin; St. Louis, MO, USA). Various groups of AIBC were fabricated: group A (0.5 g each of tobramycin + gentamicin/40 g PMMA), group B (1 g gentamicin/40 g PMMA), group C (1 g tobramycin/40 g PMMA), group D (5 g each of tobramycin + gentamicin/40 g PMMA), and group E (10 g tobramycin/40 g PMMA).

When the batches were created, the already mixed PMMA antibiotic was added to a liquid monomer, methyl methacrylate, in a stainless steel bowl and mixed with a stainless steel spatula at room temperature in a fume hood. It was then poured into custom-made polytetrafluoroethylene (PTFE) molds having cylindrical pores with dimensions of 6.0 mm × 12.0 mm (diameter × height). Thereafter, the molds were fastened between two solid PTFE plates for 1 h. After solidification, the cylindrical molds were grated against a 240-grit silicon carbide grinding disc that removed excess material and paralleled the two surfaces of the molds. Twelve specimens were taken from each set of antibiotic loads, adding up to a total of 60 specimens across all antibiotic combinations.

The specimens were removed from the molds and checked for defects or bubbles on the surface. The imperfect specimens were discarded. Internal bubbles were also checked using a Faxitron X-ray Corporation System (Tucson, AZ, USA). The X-rays were performed at 35 kV for 1.30 min with a mammography film. Within each set of antibiotic loads (each group), four specimens were identified for each time period. Finally, before testing, the four specimens (for each group and each time period) were separated into two groups (two specimens in each group): one group was tested dry and the other wet, to study the effect of hydration.

The antibiotic in each specimen was allowed to elute in buffered saline (PBS) in a shaker for the predetermined time periods of 1, 3, and 21 days. At each time period, the specimens were retrieved and kept aside in clean, capped tubes at room temperature. For wet samples, each specimen was soaked in double-distilled (dd) H_2_O for 1 h prior to testing.

### Protocol for hardness testing

The Buehler m5103 micro-indentation machine (Lake Bluff, IL, USA) was used. The instrument was balanced and the swizzle pad was removed from the stage to create a more stable surface for indentation. The micro-indenter system was tested on an aluminum bar, and the units of measurement were set to be hard Vickers pyramid number (HV) meant for metals in the Vickers scale. The pure aluminum bar has a value of 167 HV, and values recorded had a mean of 164.2 HV. The indents were made on the flat surface of the cylindrical samples because the results were more consistent when tested against the hardness for pure aluminum.

Before tests on the bone cement were initiated, the scale was set to soft HV meant for nonmetals in the Vickers scale, and the weight was set to 500 g. The ocular was checked and calibrated. The stable block given in the Buehler m5103 micro-indenter was placed on the indentation stage, and the bone cement cylinders were placed on top of the stable block vertically. Each sample was indented for an average of seven to eight times to account for the porosity and nonuniformity of PMMA. Objective lens A (MIO 0.25/∞) was used to observe and measure the indents. The indentation machine would record the values in the Vickers scale, in units of HV, which were later converted into megaPascals using a conversion constant, 9.807. For wet testing, the specimens were placed in dd H_2_O for 1 h prior to following the method followed for dry samples.

### Statistical analysis

Statistics was done on these groups to test for correlation or a linear relationship between hardness and increased antibiotic loads and then between hardness and increased elution times. A Pearson correlation coefficient was calculated for hardness values and antibiotic loads. A Pearson coefficient of −1 (*r* = −1) indicates a perfect negative relationship: as one variable increases, the other decreases. A Pearson coefficient of 1 (*r* = 1) indicates a perfect positive relationship: as one variable increases, the other increases. The coefficients do not, on the other hand, indicate the strength of the relationship. *P* values were also calculated for correlation at a significance level of α = 0.05, with a null hypothesis of *r* = 0 and a research hypothesis of *r* ≠ 0. *P* values less than 0.05 indicated a correlation, whether positive or negative, while *p* values greater than 0.05 indicate no correlation.

## Author’s information

SS is the Director of the Biomedical Engineering Program and a professor in the School of Graduate Studies at the Department of Orthopaedic Surgery and Rehabilitation Medicine, SUNY Downstate Medical Center.

## Authors’ original submitted files for images

Below are the links to the authors’ original submitted files for images. Authors’ original file for figure 1 Authors’ original file for figure 2 Authors’ original file for figure 3
