# Supplementary figures and images for: Microhardness of bi-antibiotic-eluting bone cement scaffolds
Source: Prog Biomater. 2012 Oct 8;1:3. doi: 10.1186/2194-0517-1-3 (PMC5120661; doi:10.1186/2194-0517-1-3)

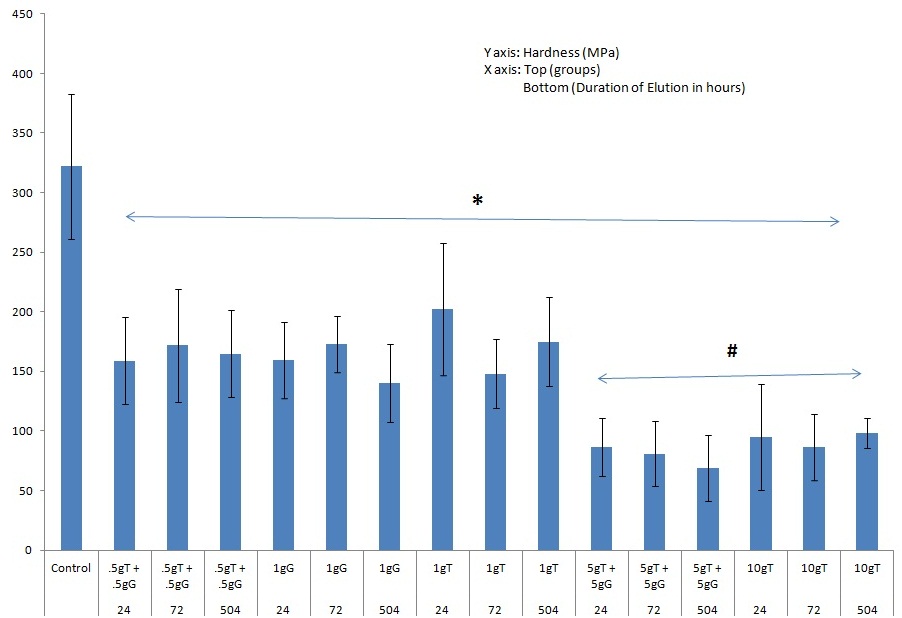

Supplement: Supplementary file 1 — Authors’ original file for figure 1 [file 40204_2012_3_MOESM1_ESM.jpeg]

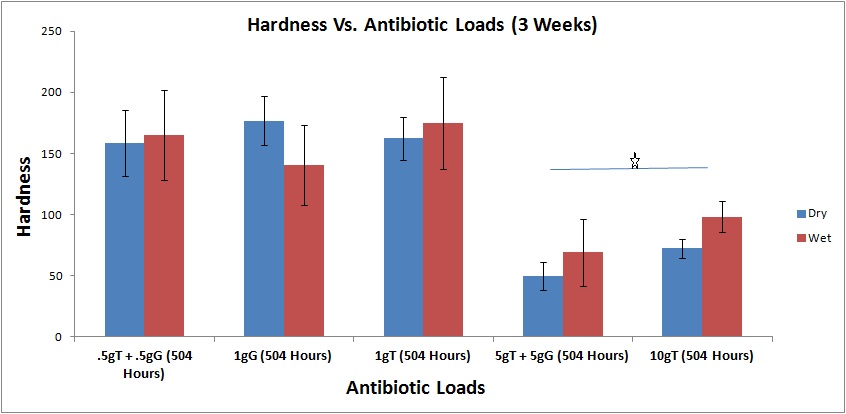

Supplement: Supplementary file 2 — Authors’ original file for figure 2 [file 40204_2012_3_MOESM2_ESM.jpeg]

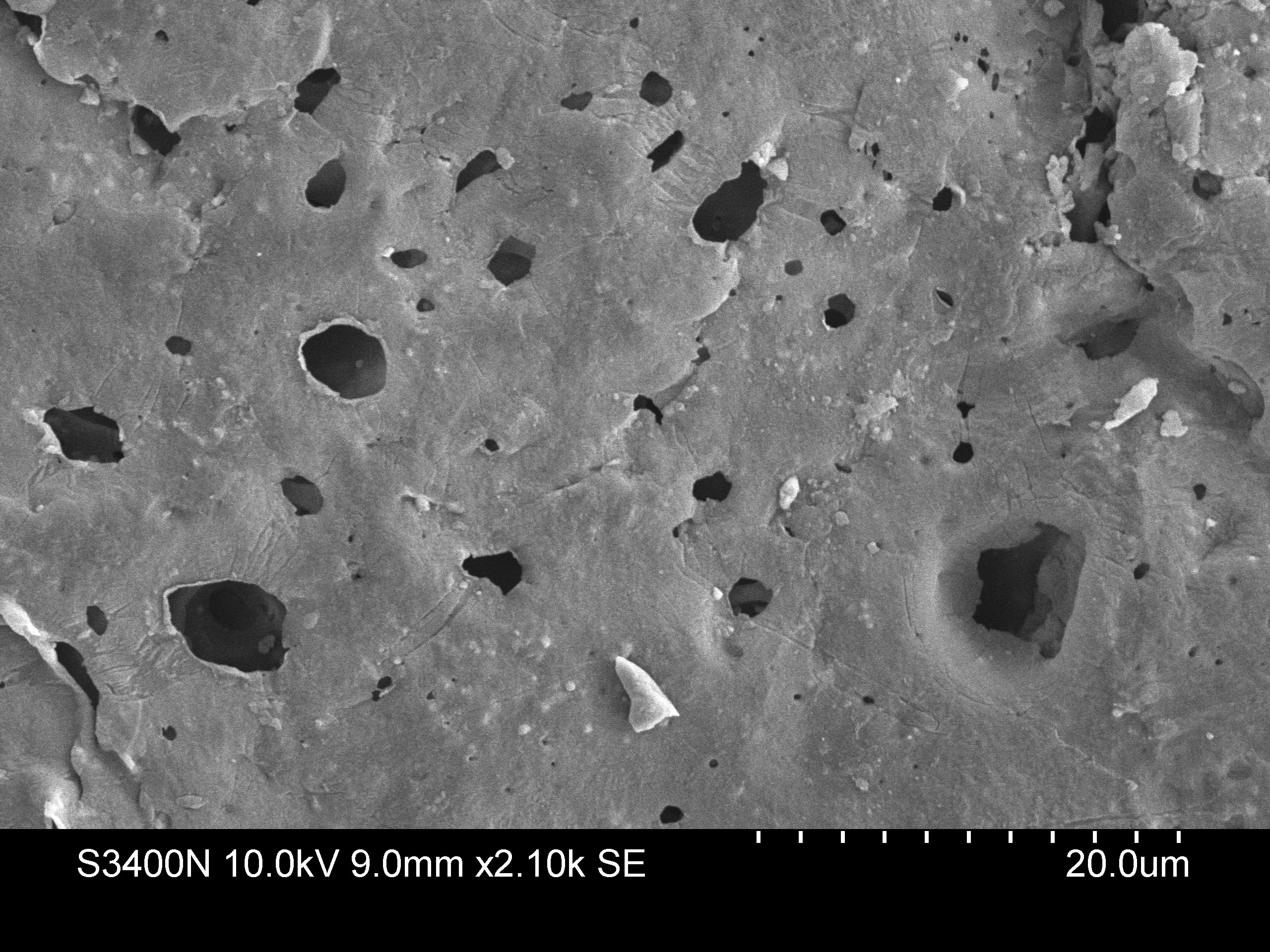

Supplement: Supplementary file 3 — Authors’ original file for figure 3 [file 40204_2012_3_MOESM3_ESM.jpeg]
